# What is the best histopathological classification for celiac disease? Does it matter? A letter of comment to the review of Amado Salvador Pena; a new proposal

**Published:** 2015

**Authors:** Vincenzo Villanacci

**Affiliations:** *Institute of Pathology Spedali Civili Brescia Italy*

**To The Editor:**

I read with great interest the excellent review of Amado Salvador Pena about the problems related to the histopathological classification of celiac disease (CD) (1). The widespread knowledge of the multiform clinical manifestations of CD, together with the availability of highly sensitive and specific serological tests such as tTG, have significantly reduced the relevance and impact of small bowel biopsy in the diagnostic approach to CD. Small bowel biopsy can no longer be viewed as the gold standard for the diagnosis of CD and it is quite likely that the next guidelines produced by gastroenterological societies worldwide will suggest that small bowel biopsy be carried out only in selected cases of suspected CD. Far from reducing the relevance of the procedure, it is important to keep in mind the importance of an accurate and standardized approach to each step, from biopsy taking to handling and processing of biopsy specimens. In this sense as

**First point**, the orientation of the biopsies is fundamental and mandatory is the use of acetate cellulose filters already cut to avoid incorrect evaluation of the architecture of villi (Figure 1); **second point** the different histopathological classifications Marsh, Marsh modified by Oberhuber, Corazza Villanacci and Ensari represent another important crucial and misleading point in this field in particular “ the greater the number of diagnostic categories of a method, the lower its diagnostic reproducibility “ .For the above reasons I propose a simplified histological scheme for the diagnosis of CD that could help the pathologists and the clinicians (2): 

Only two categories:

1. **Type** **A** normal villi but with a pathological increase of T lymphocytes.
2. **Type B **atrophy of villi with a pathological increase of T lymphocytes (Figure 2).

Two points are really important:

**The first:** in presence of normal villi the pathological increased number of intraepithelial T lymphocytes is crucial but attention! This condition is found in different pathological disorders as well and it is not specific of CD (3). 

**The second:** the presence of atrophy of villi with the concomitant pathological increase in the number of intraepithelial T lymphocytes without differentiation in the grade of atrophy.

 The above scheme presents some advantages:

- The elimination of different categories in the condition of atrophy, not useful for the gastroenterologist

A more simple approach to the initial lesions of CD; in this sense when the clinical and serological data are absent we prefer the term proposed by Rostami et al. of **Microscopic Enteritis** (4). This term is indicative of a pathological situation without the necessary elements leading to a correct aetiological diagnosis and a stimulus for the clinician to make further serological, clinical and genetic tests to arrive at a correct diagnosis.

**Figure 1:**
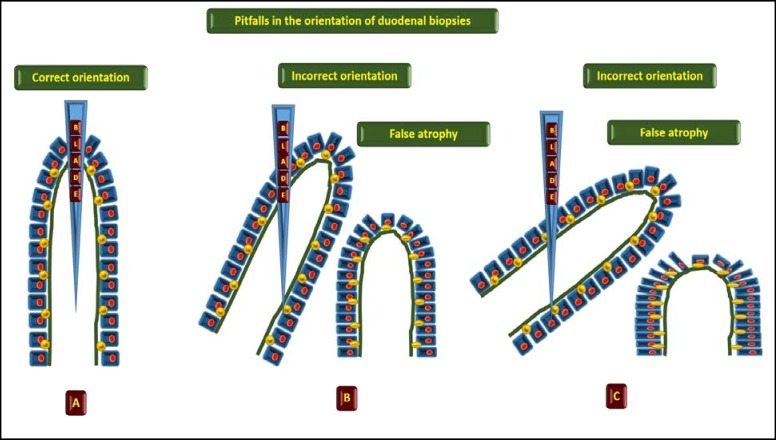
The orientation of the biopsies to avoid incorrect evaluation of the architecture of villi

**Figure 2:**
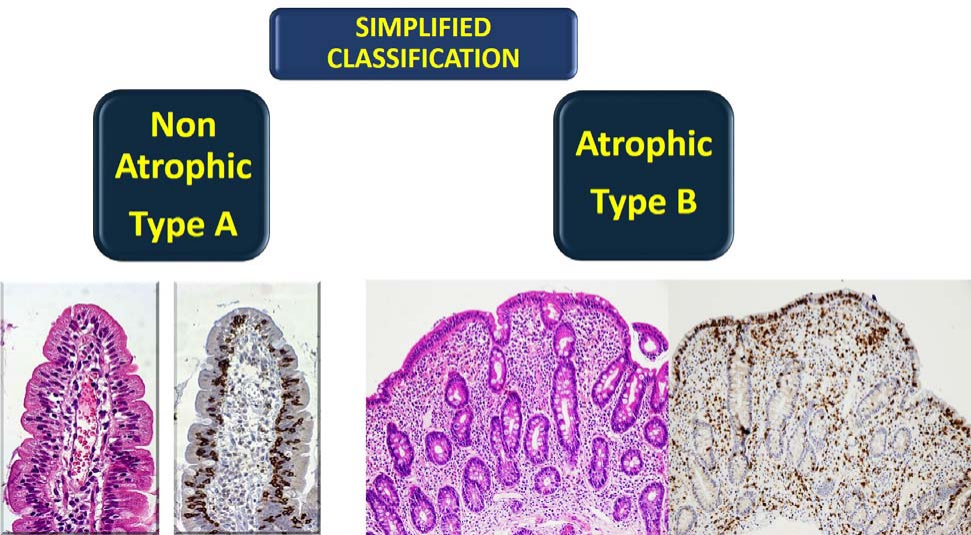
A simplified histological scheme for the diagnosis of CD

## Note

Please see the pages: 239-243
